# Morphokinetic Behavior of the Second Polar Body in Human Zygotes as a Predictor for Embryonic Developmental Potential: An Exploratory Study Based on Time-Lapse Observation

**DOI:** 10.3390/ijms26073190

**Published:** 2025-03-29

**Authors:** Toko Shimura, Panagiota Tsounapi, Keitaro Yumoto, Yasuyuki Mio

**Affiliations:** Reproductive Centre, Mio Fertility Clinic, Yonago 683-0008, Japan; tokos@mfc.or.jp (T.S.); yumotok@mfc.or.jp (K.Y.)

**Keywords:** ART, second polar body, morphokinetic behavior, time-lapse cinematography, embryo development

## Abstract

Time-lapse imaging has made possible the detailed observation of all stages of embryonic development, including also from the extrusion of the second polar body up to the first cleavage. By extensive observation, we achieved detection of a variety of behaviors of PBIIs such as (a) morphologically static behavior, (b) amoeboid movement, (c) shrinking, (d) fragmenting, and (e) ruffling. Retrospective analysis was performed on 282 ICSI zygotes derived from 69 ART treatment cycles from January to August 2019. Zygotes with morphologically static PBIIs (a) and PBIIs showing various behaviors (b)~(e) were classified into Group 1 (n = 70) and Group 2 (n = 212), respectively. Based on the rates of irregular division, good quality embryos, and the time from the PBII extrusion, pronuclear breakdown to the first cleavage was compared between groups (Study 1). Furthermore, the relationship between the type of PBII behaviors and ploidy in 94 biopsied blastocysts from 15 cycles was examined, in which one or more euploid embryos were obtained between August 2021 and July 2024 (Study 2). The results showed that good quality embryos tended to have morphologically static PBIIs, and that euploid embryos were absent in embryos with fragmenting and ruffling PBIIs. The behavior of PBIIs may be a new predictor of embryonic developmental potential, and, in the future, morphokinetic behaviors of PBIIs may be a useful parameter for AI-assisted embryo evaluation systems.

## 1. Introduction

The development and the advancement of time-lapse incubators have provided the opportunity to observe all embryonic stages, from fertilization up to the development of a blastocyst and finally hatching. What the professionals in the field of reproductive medicine and assisted reproduction aim for is to find new markers and design new criteria as least invasive as possible to distinguish the normally fertilized oocytes with a potential to develop into a good quality blastocyst from those with lower potential.

The presence of the first polar body (PBI) is the evidence that an oocyte is mature and that it has undergone its first meiotic division. Upon fertilization, the oocyte nucleus undergoes the second meiotic division, which results in half the number of chromosomes through the extrusion of the second PB (PBII). The female pronucleus (PN) makes its appearance closer to the PBII, and the male PN, which is larger in size, usually appears first, although sometimes it is difficult to differentiate.

Since the beginning of the 21st century, there has been a lot of interest on whether PBs are of any predictive value for oocyte quality, embryo quality, or embryo euploidy. In the beginning, the focus was on the PBI, with Ebner and coworkers demonstrating that oocytes with intact PBIs were less likely to have extended fragmentation on day 2, were more likely to become blastocysts, and had higher implantation rate compared to oocytes with fragmented PBIs [1]. A year later, in 2003, Verlinsky and his coworkers, analyzing a larger number of oocytes, concluded that the morphology of the PBI might not be a useful predictive marker of developmental potential of chromosomal normality [2]. Biswas and Sang-Hwan [3] as well as Navarro and coworkers [4] brought back to discussion the predictive value of the PBI after analyzing 3177 metaphase oocytes that were fertilized by intracytoplasmic sperm injection (ICSI). Their results demonstrated that oocytes with enlarged PBI had significantly lower fertilization rate, cleavage rate and good-quality embryo rate compared to oocytes with normal size intact PBI or fragmented [3,4]. All the information gained from these studies is very valuable for present and future research, but the morphology of the PBI can practically be of use only if mature oocytes can be normally fertilized.

In the present study, we focused on the PBII and the events that take place from fertilization up to blastocyst formation. Through careful observation, we noticed that after its extrusion, the PBII forms several shapes. This led us to observe in more detail the different morphologies and the morphokinetic behaviors of the PBII and to further categorize them into the following: (a) morphologically static, (b) demonstrating amoeboid movement, (c) gradually shrinking, (d) fragmenting, and (e) ruffling. In the present study, the various morphokinetic behaviors of the PBII were investigated in correlation with the subsequent embryonic development and the ploidy of the embryos.

## 2. Results

### 2.1. Study 1

#### 2.1.1. Zygote Distribution According to Their PBII Morphokinetic Behavior

**Figure 1:**
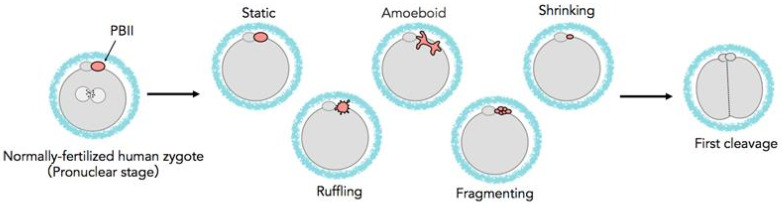
Schematic diagram of various behaviors of the PBII.

Figure 1 shows the different types of morphokinetic behaviors of the PBII. Table 1 presents the distribution of the zygotes according to the morphokinetic behaviors of their PBIIs into two groups. Group 1 included the zygotes with morphologically static PBIIs, and Group 2 included zygotes with various PBII behaviors. The proportion of the zygotes allocated into Group 1 was 24.8% (n = 70/282) and into Group 2 was 75.2% (n = 212/282). Interestingly, PBIIs demonstrating amoeboid movement was the most frequent (42.6%, n = 120/282). However, considering the changes along the time axis, PBII behaviors of Groups 2 embryos were manifold.

The PBIIs were observed and categorized, according to their morphology and kinetic behaviors, into the following categories: a. static, where the PBII appears round in shape and relatively still; b. amoeboid, where the PBII appears in irregular shape with movement similar to amoebas, extending and crawling; c. shrinking, where the PBII slowly grows smaller in size, d. ruffling, where the PBII appears like a circle with many extensions around it, expanding and contracting; and e. fragmenting, where the PBII gradually breaks into fragments.

#### 2.1.2. Patients’ Background and Embryonic Development Outcomes

Table 2 presents the clinical outcomes of the embryonic development. The mean maternal age was similar among the groups, 34.8 ± 5.4 y.o. in Group 1 and 35.3 ± 5.0 y.o. in Group 2, with no significant difference. The incidence of irregular (direct/reverse) divisions at the first cleavage showed no significant difference between the two groups (21.4% in Group 1 and 28.3% in Group 2). Good quality embryos at the early cleavage stage (Day-2 embryos) were cryopreserved or transferred on Day-2/3 according to our clinic’s policy, and only those that were not cryopreserved or freshly transferred on Day-2/3 were extendedly cultured up to Day-7. In Group 1, 33 early embryos were cryopreserved (n = 27) or freshly transferred (n = 6) and in Group 2, 70 embryos were cryopreserved (n = 59) or freshly transferred (n = 11). As a result, 37 embryos were cultured up to Day-7 in Group 1 and 142 embryos in Group 2. The rate of good quality embryos at the early cleavage stage was significantly higher in Group 1 than in Group 2 [58.6% (n = 41/70) and 35.4% (n = 75/212), respectively; *p* < 0.01]. The rate of good quality blastocysts was 32.4% (n = 12/37) in Group 1 and 22.5% (n = 32/142) in Group 2, and there were no significant differences between the two groups. On the other hand, the utilization rate for cryopreservation and embryo transfer was significantly higher in Group 1 compared to Group 2 [64.3% (n = 45/70) and 48.1% (n = 102/212), respectively; *p* < 0.05].

#### 2.1.3. The Sequential Events from PBII Extrusion up to the First Cleavage

**Figure 2:**
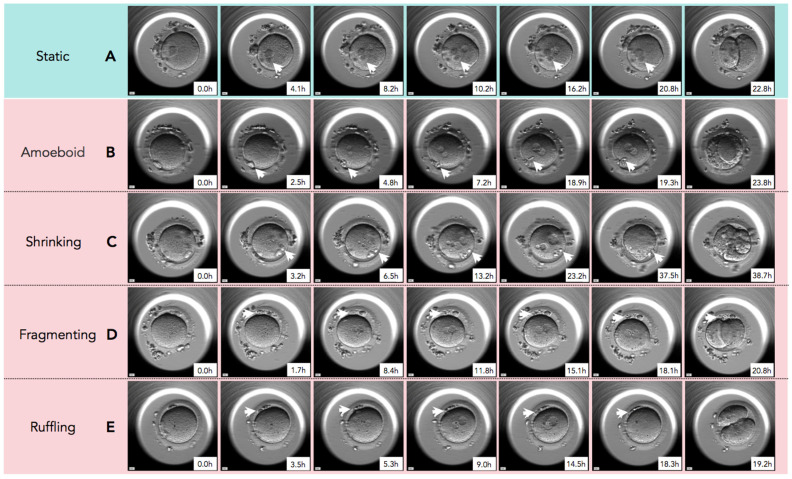
The sequential still images of zygotes from the time of second polar body (PBII) extrusion to the first cleavage. (**A**) Static: PBII rarely moved and its shape did not change from the time of extrusion to the first cleavage. (**B**) Amoeboid: PBII moved and changed its shape like amoeba after the extrusion to the first cleavage. (**C**) Shrinking: The size of PBII just before the first cleavage was smaller than immediately after the extrusion. (**D**) Fragmenting: A round-shaped PBII gradually fragmented over time. (**E**) Ruffling: The shape of PBII ruffled during the observation. White arrowheads indicate the PBII. Magnification:.

**Figure 3:**
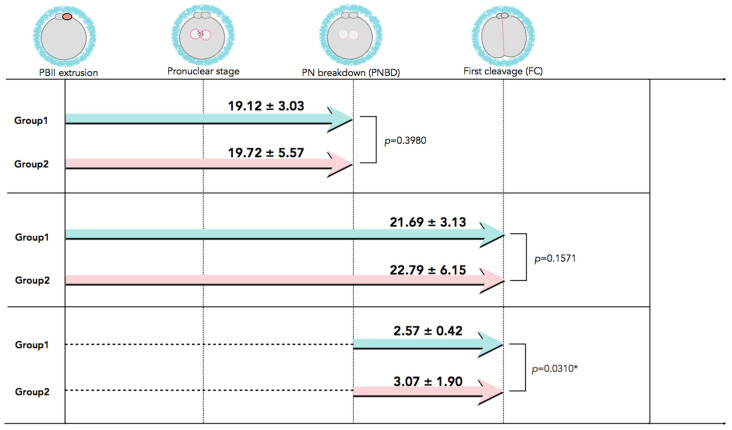
The developmental events before the first cleavage with time interval values. Although there was no significant difference in the time interval from PBII extrusion up to the first cleavage between the two groups, the time interval from PNBD to the FC was significantly longer for Group 2 than for Group 1. Data are presented as mean ± SD in hours. * Statistical significance: *p* < 0.05. FC: first cleavage; PBII: second polar body; PNBD: pronuclear breakdown.

In Figure 2, time-lapse still images show developmental events, from the extrusion of the PBII up to the first cleavage, for all five categories of PBII morphokinetic behaviors. Supplemental Videos S1–S5 are also provided for more detailed observation. Figure 3 presents the time interval of the developmental events before the first cleavage in the two groups. Setting the extrusion of the PBII as the baseline, the time up to the pronuclear breakdown and the time up to the first cleavage showed no significant differences between the two groups. There were no significant differences among the groups on the time of PBII extrusion (Table 3). Interestingly, the time interval from pronuclear breakdown up to the first cleavage was significantly shorter in Group 1 (2.57 ± 0.42 h) compared to Group 2 (3.07 ± 1.90 h).

#### 2.1.4. Sperm Parameters

Table 4 presents the results of the semen analysis of the male partners in Group 1 and Group 2. There were no significant differences in the sperm parameters between the two groups.

### 2.2. Study 2

#### The PGT-A Results

**Figure 4:**
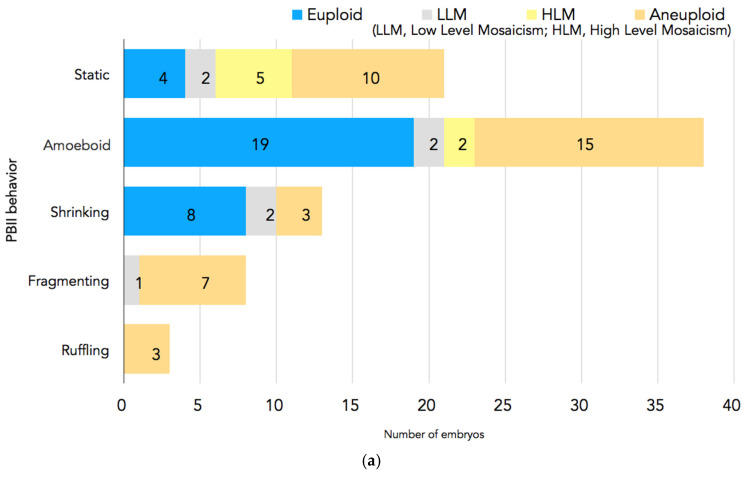

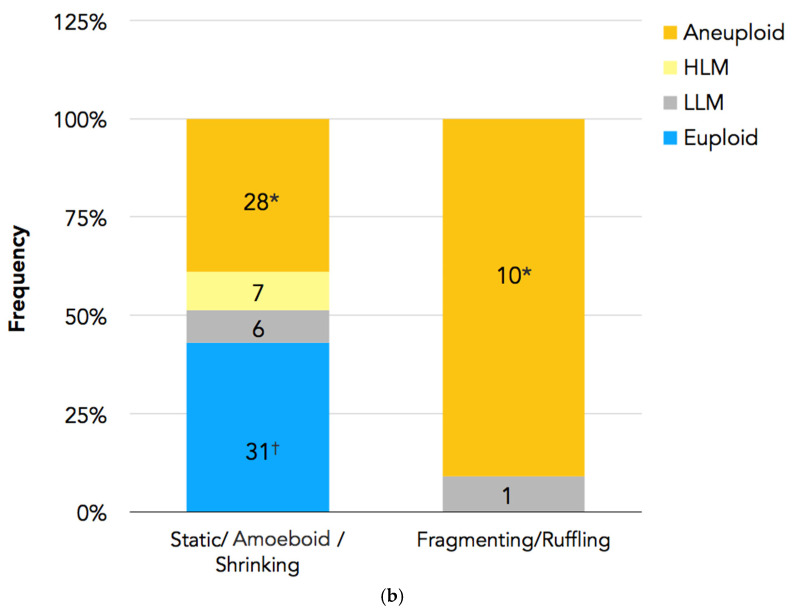
(**a**) PGT-A results for each PBII morphokinetic behavior. Embryos with static PBIIs: 4 euploid embryos, 2 embryos with low-level mosaicism, 5 embryos with high-level mosaicism, and 10 aneuploid embryos. Embryos with amoeboid PBIIs: 19 euploid embryos, 2 embryos with low-level mosaicism, 2 embryos with high-level mosaicism, and 15 aneuploid embryos. Embryos with shrinking PBIIs: 8 euploid embryos, 2 embryos with low-level mosaicism, and 3 aneuploid embryos. Embryos with fragmenting PBIIs: 1 with low-level mosaicism and 7 aneuploid embryos. Embryos with ruffling PBIIs: 3 aneuploid embryos. (**b**) The number of euploid embryos in each group based on the PBII behaviors. There were 31 euploid embryos with PBIIs showing static, amoebic, and shrinking behaviors (37%; n = 31/72), whereas there were no euploid embryos with ruffling and fragmenting PBIIs (0%; n = 0/11). (* *p* = 0.00126, ^†^ *p* = 0.00596). The numbers of embryos are shown in the bars. PBII: second polar body.

In Figure 4a,b, the PGT-A results are presented. In total, 94 embryos had undergone biopsy. Nine embryos were excluded because there were no clear images of the PBII, or the PBII was unidentifiable; two embryos were excluded because they did not have results. Therefore, 83 biopsied embryos with suitable PBII images were analyzed. Among the embryos with fragmenting or ruffling PBIIs, there were no euploid embryos. Meanwhile, among the embryos with static, amoeboid, or shrinking PBIIs, there were 31 euploid embryos.

## 3. Discussion

To our knowledge, this is the first study in the international literature where the morphokinetic behaviors of the PBII have been extensively investigated and the relationship with embryo development has been analyzed. Our results demonstrate that zygotes with morphologically static PBIIs have significantly higher rates of good quality Day-2 embryos and higher utilization rates (embryos that were cryopreserved plus the embryos that were freshly transferred) compared with the zygotes that demonstrated various morphokinetic behaviors of the PBII. Additionally, in order to investigate the relationship between the morphokinetic behaviors of the PBII and euploidy, we found that embryos with static, amoeboid, or shrinking PBIIs were more likely to be euploid compared to the embryos with fragmenting or ruffling PBIIs where there were no euploid embryos.

The number of studies that had somehow utilized the PBII in order to evaluate the quality of the oocytes or embryos is very limited. Previously, Kim and coworkers demonstrated that the angle of the spindle was correlated with the angle between the PN axis and the PBII and that it has an effect on embryo quality [5]. Additionally, the same research group showed that the smaller the angle between the spindle and the PBII is, the higher the possibility that an embryo is euploid [6]. Of course, these are preliminary data, and further research is necessary to shed more light in the relationship between the oocyte spindle/PBII angle and euploidy.

In 2022, Yang and coworkers investigated whether there is a correlation between the angle of the two PBs, the status of the two PBs, and embryonic euploidy [7]. The authors, according to the size of the angle between the two PBs, made three classifications, ≤30°, 30–90°, and ≥90°. Moreover, they further classified the state of the two PBs based on whether they were spherical or fragmented or irregular in shape. According to their results, the size of the two PB angles and the PB status were not directly associated with embryo euploidy, aneuploidy, or mosaicism, and they suggested that selection of euploid embryos should not be made based on these parameters [7].

Studies have shown that it is possible for the PBII to remain up to the blastocyst stage, but eventually both PBs will degenerate. Therefore, by extracting both PBs as material for PGT-A and genetically analyzing them can provide us with information for both aneuploidy and maternal mutations and is a less invasive PGT method [8].

Additionally, as far as the utilization of the PBs as material for PGT-A is concerned, there has been extensive research in this field. In the ESHRE consortium for good practice recommendations for PB and embryo biopsies for PGT, PB biopsies may be an alternative to embryo biopsies, due to regulations that prohibit embryo biopsies after the syngamy in specific regions or countries, or if only maternal pathogenic variants, structural rearrangements, or aneuploidies are investigated [9]. On the other hand, mitotic errors, paternally derived meiotic errors, and pathogenic variants cannot be detected from PBs. Moreover, removing the PBs from zygotes for PGT may be the least invasive method for the embryo, but there is no guarantee that that the zygotes will become good-quality early embryos or blastocysts compatible for embryo transfer. Nonetheless, in case of maternally derived meiotic aneuploidies or maternal pathogenic variants, this biopsy strategy is sufficient for testing. The amount of DNA is limited, because single cells are analyzed, and the estimated rate of inconclusive diagnosis is expected to be lower than 10% [9].

According to the Cohrane review analysis, there was not enough evidence to determine whether there is any improvement in the cumulative live birth rate or live birth rate after the first embryo transfer with the addition of PGT-A by PB biopsy [9]. On the other hand, the study showed that there might be a reduction in the miscarriage rate with the addition of PGT-A by PB biopsy [10].

For the utility of PBs in basic research for assisted reproduction, another study in mouse embryos has provided valuable information on the importance of the PBII. Gardner in 2000 showed that almost always, the PBII persists intact to the early blastocyst stage [11]. In that study, Gardner demonstrated that the PBII is attached during all stages to the conceptus via a thin, extensible, weakly elastic cord, and can remain coupled through ions up to the blastocyst stage [11]. These findings are in agreement with the study by Jin and coworkers, where the critical role of the PBII in the asymmetric fate of the mouse embryo was demonstrated for the first time [12]. The authors showed that the PBII, through material exchange with the zygote, may help to break the symmetry of the zygote, endowing the blastomere with the PBII attached to it with inner cell mass (ICM) lineage preference. Moreover, the authors suggested the potential roles of the PBII in embryonic development. Since the PBII and the zygote are physically connected, there is a possibility of material exchange between them. By immunofluorescence experiments, they showed that proteins could transport from both directions between the PBII and the zygote during the period of the first cleavage, and probably this exchange is ended at the two-cell stage. Additionally, they showed that the speed of this protein transport is faster from the PBII to the zygote than from the zygote to the PBII [12].

Taking into consideration that the results from the studies by Gardner 1997 [11] and Jin et al. 2022 [12] in mouse embryos showed that there is a kind of communication and exchange of materials between the PBII and the zygote, we cannot exclude the possibility that also in the human zygote, the PBII may have some kind of translational and transcriptional activity from fertilization up to the two-cell stage. This could partially explain the fact that in our study, the rate of good-quality Day-2 embryos was significantly higher in Group 1 compared to embryos in Group 2. The stability of the PBII can let the material exchange take place in a more appropriate way, and the early cleavages may result in better-quality early embryos with less fragmentation and, furthermore, significantly higher utilization rates compared to the zygotes that demonstrated various morphokinetic behaviors. Focusing on the PGT-A results in our study, it was interesting that among the embryos with amoeboid or shrinking PBIIs were euploid embryos. On the other hand, the embryos with ruffling or fragmenting PBIIs did not have any euploid embryos. Taking a closer look at the differences between these various morphokinetic behaviors of the PBII, we can see that in amoeboid and shrinking PBIIs, the physical connection with the zygote remains, as opposed to ruffling or fragmenting PBIIs. A possible explanation could be that from the second meiotic division and subsequently the extrusion of the PBII, up to the first cleavage, in the case of the fragmenting or ruffling PBII, there are some events that did not allow the equal segregation of the bivalents, resulting in aneuploidies. This may be explained by the fact that in the fragmenting or ruffling PBIIs, the physical contact between the zygote and the PBII is somehow interrupted. The lack of physical contact could have a negative impact in the necessary exchange of transcriptional or translational material, because of defects that might have happened during the second meiotic division in the first place, and this is why the PBII demonstrates this morphokinetic behavior (fragmenting or ruffling). Of course, these are speculations, and we need immunofluorescence experiments using live cell imaging in order to confirm them. However, considering the restrictions and ethics that rule the legislations for human embryos, it is very difficult to perform such experiments.

One of the limitations of the present study may be that the number of the zygotes allocated in each group according to the evaluation of the PBIIs is unequal. In the future, we need to increase the number of analyzed zygotes in order to make our criteria stronger, as well as observe more carefully whether there are changes in the morphokinetic behaviors of the PBII over time up to the first cleavage stage.

## 4. Materials and Methods

### 4.1. Ethics Approval

The present study was approved by the Ethics Committee and Institutional Review Board of Mio Fertility Clinic, and approval for publication of this study was requested and granted (20230420-001). Patients provided permission for their data to be analyzed with full informed consent. No identifiable patient information is included in this report.

### 4.2. Patients and Experimental Design

#### 4.2.1. Study 1

**Figure 5:**
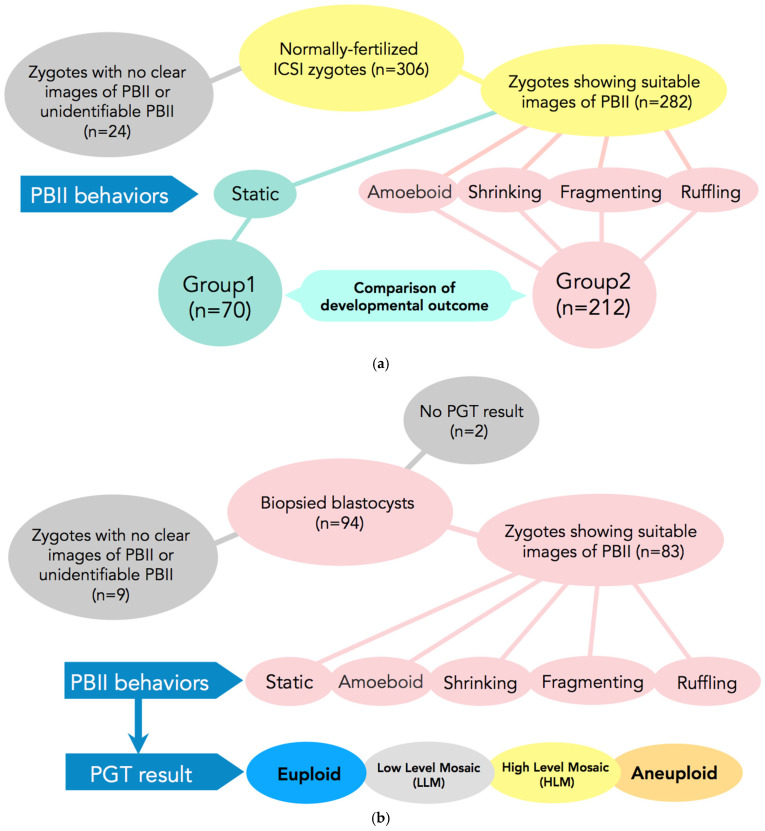
(**a**) Participants and setting of Study 1. In total, 306 normally fertilized ICSI-zygotes were obtained. Of those, 24 were excluded from the study due to difficulties in observing the PBII. A total of 282 zygotes were included in the study. These 282 zygotes were allocated into two groups according to the morphokinetic behavior of the PBII. In Group 1 zygotes with static PBII were included (n = 70) and in Group 2 the remaining 4 types of PB II were included (n = 212). ICSI: intracytoplasmic sperm injection; PBII: second polar body. (**b**) Subjects and setting of Study 2. Ninety-four blastocysts that had undergone biopsies were selected for analysis. Of those, nine were excluded due to difficulties in identifying the PBII and another two due to no results. A total of 83 blastocysts were included for analysis from the zygote stage up to becoming a blastocyst suitable for biopsy. The 83 zygotes were allocated into five categories according to the behaviors of the PBII (static, amoeboid, shrinking, fragmenting, and ruffling). The blastocysts after the PGT results were allocated as euploid, low-level mosaic, high-level mosaic, and aneuploidy.

The present study is a retrospective study that included 306 normally fertilized zygotes by ICSI from time-lapse imaging from January 2019 to August 2019. Of those, 282 zygotes demonstrated suitable images for PBII analysis from extrusion up to the first cleavage (Figure 5a).

Zygotes with morphologically static PBIIs were classified in Group 1 (n = 70) and zygotes with various morphokinetic behaviors (amoeboid, shrinking, fragmenting, and/or ruffling) were included in Group 2 (n = 212).

#### 4.2.2. Study 2

Based on the results of Study 1, we further examined whether there is any relationship between the morphokinetic behavior of the PBII and embryo euploidy. For this purpose, 94 blastocysts that underwent biopsies were selected from 15 cycles for analysis, from August 2021 up to July 2024. Of those, two did not have available results from the preimplantation genetic testing for aneuploidy (PGT-A), and nine blastocysts did not have PBII images suitable for analysis. In total, 83 embryos satisfied the criteria and were included in the study. Fertilization of the selected blastocysts was achieved either by conventional in vitro fertilization (c-IVF) or ICSI. As selection criteria, we set as a necessary condition that the cycles must include at least one euploid blastocyst (Figure 5b).

### 4.3. Ovarian Stimulation and Oocyte Aspiration

Ovarian stimulation for ovum pick-up (OPU) was performed according to a combination of GnRH analog and gonadotropin treatment protocols. The administration of human menopausal gonadotropin (Folyrmon-P; Fuji Chemicals Industrial, Toyama, Japan) started on the third day of the cycle (after taking into consideration the age and anti-Müllerian hormone (AMH) level of each patient), and luteinizing hormone-rich menopausal gonadotropin was administered (HMG Ferring; Ferring Pharmaceuticals, Saint Prex, Switzerland) from the middle of the cycle until the day of human chorionic gonadotropin (hCG) administration. For GnRH analog treatment, we started nasal administration of a GnRH agonist (nafarelin acetate hydrate, Nasanyl 0.2%; Pfizer Inc., New York, NY, USA) on the first day of menstruation. The follicular development was monitored every other day by measuring serum concentrations of estradiol, progesterone, and luteinizing hormone, as well as transvaginal ultrasonography from the seventh day of the menstrual cycle until hCG was administered for triggering ovulation. When the leading follicle reached a three-dimensional diameter of 20 mm, 10,000 international units of hCG (HCG MOCHIDA; Mochida Pharmaceutical, Tokyo, Japan) was administered for final follicular maturation. In addition, in cycles combined with a GnRH antagonist, a GnRH agonist (1800 µg/day) was used for triggering ovulation instead of hCG to avoid ovarian hyperstimulation syndrome for patients with high levels of AMH whose serum estradiol levels were greater than 3000 pg/mL and with more than ten developing follicles larger than 15 mm in diameter. For the patients with less than 10 developing follicles and estradiol levels less than 3000 pg/mL that used GnRH antagonist, they were administered 10,000 international units of hCG for the final follicular maturation. The day before the OPU, 0.7 mL of insemination medium (Sydney IVF Fertilization^®^; COOK Medical, Bloomington, IN, USA) was dispensed into each well of a four-well dish (NM-CD-4, Nakamedical Inc., Tokyo, Japan), covered with mineral oil (WO100; Nakamedical Inc.), and equilibrated at 37 °C in a bench-top incubator (MINC^®^; Cook Medical Australia, Brisbane, Australia) under the gas phase (mixture of 5% O_2_, 6% CO_2_, and 89% N_2_) overnight.

OPU was performed 36 h after triggering ovulation using an 18-gauge oocyte aspiration needle (PTC needle; Hakko, Nagano, Japan) under transvaginal ultrasonography guidance.

### 4.4. Semen Treatment

Sperm samples were purified and adjusted to a concentration ≤ 10 × 10^6^ spermatozoa/mL by the combined method of centrifugation on density gradients of Percoll (40%, 70% and 90%; 1500 rpm, 30 min), followed by swim-up using insemination medium (insemination medium; Nakamedical Inc., Tokyo, Japan).

### 4.5. Oocyte Denuding Method and ICSI Procedure

The patients that were included in Study 1 had either severe male factor infertility or the possibility of fertilization failure (split cycles: IVF/ICSI); therefore, ICSI was applied as their treatment method. For denudation, the cumulus cells were gently removed with hyaluronidase (ICSI Cumulase; Origio, Måløv, Denmark) and pipetting in HEPES-buffered medium (HEPES, Nakamedical Inc., Tokyo, Japan) 1 h after oocyte retrieval. Denuded oocytes were transferred into a drop of the equilibrated insemination medium prepared in a two-well dish (Nakamedical Inc., Tokyo, Japan) and cultured in a MINC^®^ incubator for at least 3 h before sperm injection. Then, ICSI was applied to mature oocytes based on the method described by Payne et al. [13]. Oocytes after ICSI were immediately placed in a Geri Dish or EmbryoSlide with equilibrated sequential medium and cultured in Geri (Geri Dish; Genea Biomedx, Sydney, Australia) or Embryoscope^®^ (Vitrolife, Göteborg, Sweden), respectively.

### 4.6. c-IVF Procedure

Retrieved oocytes were placed into insemination medium (insemination medium; Nakamedical Inc., Tokyo, Japan) in a 4-well dish (Nakamedical Inc., Tokyo, Japan) and pre-cultured in a MINC^®^ bench-top incubator for 1 to 4 h. Subsequently, they were inseminated with approximately 50,000 motile spermatozoa per oocyte and co-cultured in a MINC^®^ bench-top incubator. Five hours post-insemination, the oocytes were denuded mechanically by pipetting and transferred to a specially designated dish (Geri Dish; Genea Biomedx, Sydney, Australia or EmbryoSlide; Vitrolife, Göteborg, Sweden) with sequential medium (Sydney IVF cleavage medium; COOK Medical) equilibrated under the mixed gases and cultured under a time-lapse monitoring system and incubator (Geri^®^; Genea Biomedx Sydney, Australia & Embryoscope^®^; Vitrolife, Göteborg, Sweden).

### 4.7. Morphological Evaluation of Embryos

Early-stage embryos were evaluated based on Veeck’s criteria modified according to the Istanbul Consensus [14,15]. The 2PN zygotes examined in this study were divided into three categories based on the amount of cytoplasmic fragmentation and the size of blastomeres: category 1 (Grade 1 based on modified Veeck’s criteria, containing ≤ 20% fragments), category 2 (Grade 2, >20–<40% fragments), and category 3 (Grade 3, ≥40% fragments). In addition, a developed blastocyst was defined as the stage when the formation of the blastocoel was confirmed. A morphologically good-quality blastocyst was defined as a blastocyst graded as ≥4BB according to Gardner’s criteria [16]. Embryo evaluation based on the definitions and criteria above was conducted by at least two senior embryologists.

### 4.8. Blastocyst Biopsy Procedure

For blastocysts of full expansion that did not achieve hatching on Day 5, 6, or 7, assisted hatching was applied. In brief, expanded blastocysts were placed in drops of 0.25 M trehalose in a 60 mm dish (Thermo Fisher NUNC, Waltham, MA, USA) covered with heavy oil and were fixed with a holding pipette (Sankyo Medics, Shizuoka, Japan). Subsequently, a laser (LYKOS Laser; Hamilton Thorne, Beverly, MA, USA) was used to irradiate areas of the zona pellucida (ZP) where there was sufficient space between the ZP and the trophectoderm, in order to create a small opening with a diameter of approximately 30 μm in the ZP to allow the trophectoderm cells to herniate. Depending on the response to the assisted hatching of each blastocyst (varying from 30 min up to few hours) after the opening in the ZP, 5–8 trophectoderm cells were gently aspirated into the biopsy pipette (TIP-BP; The Pipette Company, Adelaide, Australia & B-23-30; ICSION, Thebarton, Australia) followed by laser-assisted cutting and flicking the holding pipette in order to remove the biopsy sample from the rest of the blastocyst. The obtained trophectoderm cells were washed in sterile PBS, then placed in microtubes containing PBS and PVP, and placed in a freezer until they were sent to the OVUS laboratory (Nagoya, Japan) for analysis. Subsequently, the blastocysts were vitrified, cryopreserved, and stored in liquid nitrogen tanks.

### 4.9. Statistical Analysis

Statistical analysis was performed using two-tailed Student’s *t*-test and chi-square test. Statistical differences were considered significant at a *p* value smaller than 0.05 (<0.05). Sample sizes and *p* values are all presented in tables or figures, where applicable.

## 5. Conclusions

This is the first time demonstrating that there might be a relationship between the morphokinetic behavior of the PBII and the embryonic development potential or euploidy.

The present study reveals that zygotes with morphologically static PBIIs are more likely to develop into a good quality embryo that can be utilized, meaning to be transferred or cryopreserved, compared to zygotes with PBIIs showing various morphokinetic behaviors. Additionally, our findings indicate that euploid embryos are absent among embryos with ruffling or fragmenting PBIIs. The behavior of PBIIs may be a new indicator for embryo assessment, but further clarification is needed for clinical use.

## Figures and Tables

**Table 1:** Rates of embryos with PBIIs showing various behaviors. PBII: second polar body.

	PBII Behaviors	Number of Embryos	Frequency
**Group 1**	Static	70	24.8%
**Group 2** (n = 212)	Amoeboid	149	52.8%
	Ruffling	25	8.9%
	Shrinking	21	7.5%
	Fragmenting	17	6.0%
	Total	282	

**Table 2:** Mean maternal age and clinical outcomes on embryo development and utilization rates of the embryos. Most good-quality, early cleavage-stage embryos are transferred or cryopreserved on Day-2/3 according to our clinic’s policy. * according to Gardner’s criteria. ^†^ *p* < 0.05, statistically significant.

	Group 1 (n = 70)	Group 2 (n = 212)	*p* Value
**Mean maternal age (year)**	34.8 ± 5.4	35.3 ± 5.0	0.7270
**Rate of irregular divisions** **at the 1st cleavage**	21.4% (15/70)	28.3% (60/212)	0.2591
**Rate of good quality** **Day-2 embryos**	58.6% (41/70)	35.4% (75/212)	0.0006 ^†^
Rate of good quality blastocysts * (≥4BB)	32.4% (12/37)	22.5% (32/142)	0.2130
**Utilization rate for cryopreservation or ET**	64.3% (45/70)	48.1% (102/212)	0.0188 ^†^

**Table 3:** Times of PBII extrusion from the start of time-lapse culture (*p* > 0.05, no statistical significance among the groups).

PBII Behavior	Time of PBII Extrusion (Mean ± SD, h)
Static (n = 69)	3.845 ± 1.539
Amoeboid (n = 149)	3.636 ± 1.745
Shrinking (n = 21)	4.043 ± 2.220
Fragmenting (n = 16)	3.944 ± 1.851
Ruffling (n = 25)	3.972 ± 1.447

**Table 4:** Mean sperm parameters in each group (*p* > 0.05, no statistical significance among the groups).

	Group 1	Group 2	*p* Value
**Semen Volume** **(mL)**	2.47	2.30	>0.05
**Sperm Concentration** **(×10^6^/mL)**	60.62	65.50	>0.05
**Sperm Motility** **(%)**	46.0	47.1	>0.05
**Forward Progression Score**	1.78	1.80	>0.05
**Abnormal Sperm Morphology (%)**	36.0	35.5	>0.05

## Data Availability

The original contributions presented in the study are included in the article. Further inquiries can be directed to the corresponding author.

## Supplementary Materials

The following supporting information can be downloaded at: https://www.mdpi.com/article/10.3390/ijms26073190/s1.
